# The Epileptics

**Published:** 1901-08

**Authors:** 


THE EPILEPTICS. The National Association for the Study of Epilepsy and the Care and treatment of Epileptics held its first meeting in Washington, D. C., May 14th and 15th last. Wm. Pryor Letchworth, LL. D., President, Portage, N. Y.; Dr. W. B. Spratling of the Craig Colony, Sonyea, N. Y., Secretary. The objects of the association: 1. To promote the general welfare of sufferers from epilepsy. 2. To stimulate the study of the causes and the methods of cure of this disease. 3. To advocate the care of epileptics in institutions where they may (a) Receive a common school education; (b) Acquire trades; (c) Be treated by the best medical skill for their malady. 4. To assist the various States in America in making proper provision for epileptics. Dr. W. B. Worsham, Superintendent of the State Lunatic Asylum at Austin, was present by invitation, and exhibited a splendid drawing of the proposed Texas Epileptic Institution soon to be built at Abilene, Texas; the drawings having been made under Dr. Worsham's supervision by the architect, J. L. O'Connor, of Austin; and read a paper descriptive of the same, outlining the general plan of the colony. The fact that Texas has made as a starter an appropriation of $200,000 for this grand charity—a sum more than three times as large as any appropriation made by any State must have impressed the members with some idea of how we do things in Texas. In an interview with Dr. Worsham he said: "The meeting was well attended, a number of the States having under advisement the establishment of institutions for the exclusive care and treatment of epilepsy, were represented by men who read papers giving the history of all that had been done, and what was advised along that line. Dr. Spratling, Superintendent of the Craig Colony, read a splendid paper on an "Ideal Epileptic Colony." New York was the first State to adopt the colony idea of caring for epileptics; Ohio has an institution for the epileptics, but it does not have the features of a modern colony, only a large building for the custodial care. Massachusetts has started on a very nice modern plan, but so far the institution has not developed very far. New Jersey has made a start, but on a limited scale. Texas has made the largest appropriation to begin with of any State, and it should be classed as the third State in the Union, as having fully adopted the modern colony plan, and made a substantial appropriation to start the institution. Dr. Worsham was made a member of the Executive Committee at Washington.

In an early issue of the JOURNAL we will publish Dr. Worsham's paper and a steel engraving of the Texas Epileptic Colony.

